# Bioinformatic Analyses of Whole-Genome Sequence Data in a Public Health Laboratory

**DOI:** 10.3201/eid2309.170416

**Published:** 2017-09

**Authors:** Kelly F. Oakeson, Jennifer Marie Wagner, Michelle Mendenhall, Andreas Rohrwasser, Robyn Atkinson-Dunn

**Affiliations:** Utah Department of Health, Utah Public Health Laboratory, Taylorsville, Utah, USA

**Keywords:** bioinformatics, whole-genome sequencing, next-generation sequencing, public health laboratory, infectious diseases, Utah, antimicrobial resistance, genes, genome, genomics

## Abstract

The ability to generate high-quality sequence data in a public health laboratory enables the identification of pathogenic strains, the determination of relatedness among outbreak strains, and the analysis of genetic information regarding virulence and antimicrobial-resistance genes. However, the analysis of whole-genome sequence data depends on bioinformatic analysis tools and processes. Many public health laboratories do not have the bioinformatic capabilities to analyze the data generated from sequencing and therefore are unable to take full advantage of the power of whole-genome sequencing. The goal of this perspective is to provide a guide for laboratories to understand the bioinformatic analyses that are needed to interpret whole-genome sequence data and how these in silico analyses can be implemented in a public health laboratory setting easily, affordably, and, in some cases, without the need for intensive computing resources and infrastructure.

Next-generation sequencing (NGS), also known as high-throughput sequencing, has affected many fields in the study of biology but has dramatically changed the field of genomics by enabling researchers to quickly sequence whole microbial genomes, profile gene expression by sequencing RNA, examine host–pathogen interactions, and study the vast microbial diversity in humans and the environment (*1*). Despite the benefits of NGS over traditional Sanger sequencing methods, public health laboratories (PHLs) have been slow to implement this revolutionary technology. According to the Association of Public Health Laboratories, no PHLs had NGS capabilities before 2010 (*2*). The Centers for Disease Control and Prevention (CDC), through its Advanced Molecular Detection program, has supported the adoption of NGS and whole-genome sequencing (WGS) by providing funding and training to PHLs. By the end of 2015, CDC’s support had enabled 37 PHLs to acquire NGS instrumentation, with another 9 PHLs gaining NGS technology by the end of 2016 (*2*).

For laboratory surveillance of foodborne diseases, pulse-field gel electrophoresis (PFGE) is currently the preferred method for typing bacterial isolates and is widely used in outbreak investigations and source tracking. PFGE has been the backbone of the success of CDC’s PulseNet program since 1997 (*3*,*4*). However, the PulseNet program is aiming to replace PFGE with WGS by 2018. This trajectory resembles the path taken in the study of human genetics, in which genetic mapping based on restriction fragment length polymorphism was replaced by quasi-complete information obtained by high-throughput genomic sequencing. Although restriction fragment length polymorphism markers initially enabled the measurement of genetic distance and laid the foundation for linkage mapping, its success depended on pronounced phenotypic effects of the underlying trait and regularly dispersed markers. Once linkage to a region was identified, causality could be pinpointed through fine mapping. WGS provided not only a complete marker-map with maximum resolution at the nucleotide level but also enabled the deduction of causality and direct testing of genetic relatedness and genetic origination. The promise of this approach also extended to the study of pathogens, given that WGS ultimately enables testing of specific hypotheses regarding genotype-phenotype relationships (e.g., antimicrobial drug resistance). However, although more PHLs are adopting NGS and WGS, only a small number of these laboratories have the ability to perform the bioinformatic analyses needed to take full advantage of the data they are generating. CDC aids PHLs in conducting foodborne disease surveillance on a national scale but is unable to assist with data analysis for local foodborne disease surveillance.

Some of the obstacles preventing PHLs from implementing the bioinformatic-dependent analysis are the requirements for large-scale computational capabilities, complex molecular evolutionary analyses, and dedicated bioinformatics staff to perform these analyses. However, all that is really needed is a computer with a browser and a connection to the Internet. Web-based tools are available for PHLs that are looking to participate in WGS data analysis but are not ready to perform analyses in-house. Several of these tools are open-source (i.e., free of charge) and can be used to perform a range of bioinformatics analyses. Two of these tools are Illumina’s BaseSpace Sequence Hub (Illumina, Inc., San Diego, CA, USA) and the Galaxy web-based platform (*5*). 

Because many PHLs are already using Illumina’s MiSeq sequencing platform, BaseSpace is a convenient solution that enables users to monitor the progress of sequencing runs, share data easily with others, and use 1 terabyte (TB) of data storage free of charge. Illumina provides new users with a 30-day free trial of BaseSpace, enabling users to use all of the wide-ranging bioinformatic tools available. 

The Galaxy platform enables users to perform analyses ranging from sequence quality control and timing to whole-genome assemblies (*5*). Galaxy also enables users to track the details of each step of an analysis, making it easier to reproduce and publish the results. Galaxy enables nonexperts to perform advanced and computationally intensive analyses without having training in bioinformatics. 

However, neither BaseSpace nor Galaxy is without drawbacks. Uploading or downloading the large files generated by NGS can be slow and might fail before finishing, requiring the entire upload or download process to be restarted. Web-based tools can also be “black boxes” where users may not know exactly what each step of the analysis is, why that step is being performed, or why results might be difficult to understand or interpret. These web-based tools might seem quick and easy to use but often do not perform as expected.

Bioinformatic analyses are often performed in a step-wise manner, with the output of 1 analysis being used as the input for the next. These multistep, multisoftware analyses are frequently referred to as pipelines and are often set up to run automatically from 1 step to the next without input from the user. In this perspective, we describe the bioinformatic pipeline implemented at the Utah Public Health Laboratory (UPHL) to analyze the WGS data. Sharing our experiences with this pipeline will enable PHLs to implement their own pipelines by following each step in our pipeline or by using our pipeline as a template to construct their own unique processes. All the software used in our bioinformatics pipeline are open-source and are available free of charge (Technical Appendix Table 1). We present these analyses as a function of the level of technology required, spanning everything from basic quality control performed on typical desktop or laptop computer to complex molecular evolutionary analyses that require powerful high-end Linux servers or workstations.

## Bioinformatic Pipeline

The bioinformatic pipeline developed and implemented at UPHL consists of 8 steps (Figure): 1) read quality control, 2) reference strain determination, 3) read mapping to the reference strain, 4) single-nucleotide polymorphism (SNP) and small insertion or deletion (indel) detection, 5) de novo genome assembly, 6) genome annotation, 7) phylogenetic tree construction, and 8) phylogenetic analysis. Although such processes are standard, several software solutions are available for the respective steps.

**Figure:**
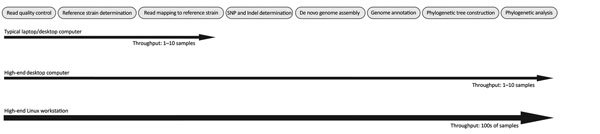
Steps in the bioinformatics pipeline implemented at Utah Public Health Laboratory.

The first step in almost all WGS bioinformatics analyses is quality control of the raw sequencing data. It is important to remove poor-quality sequence data and technical sequences (i.e., adapter sequences). Highly accurate sequences are required for SNP detection, enabling the detection of actual SNPs and distinguishing from sequencing artifacts. Quality control in our pipeline is performed by using Trimmomatic (*6*), a multithreaded command line tool that removes adapter sequences, trims low-quality sequence from the beginning or end of a sequence, removes reads that fall below a user-defined threshold for length, and validates paired-end sequence reads.

The second step in the pipeline is reference sequence determination. To determine SNPs, a reference sequence is needed against which to compare sequencing reads. The choice of reference sequence might have a substantial effect on the number and type of SNPs that are detected, making this step important. We use Mash for reference sequence determination (*7*). Mash enables us to quickly compare the large set of sequencing reads generated against the reference set of 54,118 National Center for Biotechnology Information RefSeq genomes (https://www.ncbi.nlm.nih.gov/refseq) to determine nucleotide distance and relatedness (*8*).

Once a reference sequence is determined, the next step in the analysis pipeline is mapping the quality-controlled sequencing reads to the reference genome. We perform read mapping by using the Burrows-Wheeler Aligner (BWA) software package with the bwa-mem option (*9*). BWA uses a Burrow-Wheeler Transform to efficiently align sequencing reads to reference genomes allowing for gaps and mismatches. The output of BWA is the standard sequence alignment map format known as SAM, which facilitates the next step in the pipeline.

The fourth step in the pipeline uses mapping of the sequencing reads to the reference sequence to identify SNPs and indels. We perform SNP and indel determination by using SAMtools and VarScan2, which also calculate SNP frequency in the sequence data (*10*,*11*). The output of VarScan2 can be easily viewed in the Integrative Genomics Viewer, which enables the interactive viewing of large genomic datasets (*12*). The output file of VarScan2 can also be used in more complex downstream analyses (i.e., to build SNP matrixes and phylogenetic trees).

The quality-controlled sequencing reads are then used for de novo genome assembly in the sixth step of the pipeline. We perform de novo genome assembly on individual isolates by using the St. Petersburg genome assembler, also known as SPAdes (*13*,*14*). The SPAdes assembler has 3 modules: sequencing read error correction; SPAdes assembly; and a mismatch corrector module. The first module error corrects the quality-controlled sequencing reads by using advanced algorithms based on Hamming graphs and Bayesian subclustering. Sequencing error correction in this manner has shown to dramatically improve genome assemblies of NGS data (*15*). The SPAdes assembly module uses the error-corrected reads and performs the actual assembly in an iterative manner making use of de Bruijn graphs. The resulting genome assembly is then used as input for the third module, which greatly reduces the number of mismatches and small indels by using BWA and results in highly accurate contigs (contiguous sequence data made up of overlapping sequencing reads) and scaffolds (ordered and oriented contigs based on paired-end read data).

We then annotate the resulting genome assembly to identify protein-coding genes, tRNAs, and rRNAs. We use Prokka for annotation of protein-coding genes, tRNA, and rRNA on the contigs and scaffolds generated by SPAdes (*16*). Prokka can fully annotate a bacterial genome in approximately 10 minutes on a high-end quad-core desktop computer by making use of a suite of existing software, tools, and sequence databases, such as UniProt (*17*) and NCBI RefSeq (*8*).

We then use shared orthologous genes to construct phylogenetic trees that provide insight into the relatedness of isolates. Once multiple genomes have been annotated, we calculate the pan genome of the combined genomes by using Roary (*18*). The pan genome consists of the union of genes shared by genomes of interest, and Roary can compute the pan genome of 1,000 bacterial genomes on a single CPU computer in 4.5 hours (*19*). In addition to determining the pan genome of the genomes of interest, Roary also generates a concatenated nucleotide alignment of the pan genome, which can be used to build a phylogenetic tree of these sequences. This pan genome alignment is used as the input to RAxML for phylogenetic tree construction (*20*). RAxML is a program that has been designed and optimized for conducting phylogenetic analyses on large datasets by using maximum-likelihood techniques to estimate evolutionary trees from nucleic acid sequence data (*21*).

The last step in the pipeline is phylogenetic analyses. These analyses can detect a signature of selection on individual genes and provide knowledge about the evolutionary forces acting on the genes of the sequenced isolates. The pan genome alignment can also be used to detect signatures of selection by calculating the ratio of the number of nonsynonymous substitutions per nonsynonymous site to the number of synonymous substitutions per synonymous site. The value of this ratio is used to infer the direction and magnitude of natural selection, with values >1 implying positive selection (i.e., driving change), values <1 implying purifying selection (i.e., acting against change), and values of exactly 1 indicating neutral selection (i.e., no selection). To determine the ratios for detecting signatures of selection, we use the YN00 model (*22*) implemented in the PAML software package (*23*). The PAML results are a plain text file that can be viewed in any word processor or imported into statistical analysis software, such as R, for further analysis or plotting.

## Laptop or Desktop Hardware

The bioinformatic pipeline we describe can be partitioned as a function of computer resources (i.e., the number of CPUs, the amount of RAM, and the amount of storage space). Typical laptop or desktop computers might only have enough power to perform the first steps in the pipeline, whereas a high-end workstation would have enough power to perform all the steps for hundreds of samples at once. In many cases, the limiting factor is how much RAM a computer has. Many of the more complex steps in the pipeline require large amounts of RAM, often more than what many laptops and desktops can hold. All the software described can easily be installed and run on a typical desktop or laptop computer (Figure). At UPHL, we performed steps 1–4 of the described analyses on bacterial isolates by using an Apple MacBook Pro laptop (Apple, Inc., Cupertino, CA, USA) with a single 3.2-GHz Intel Core i5 processor, 16 gigabytes (GB) of RAM, and 500 GB of storage space (Technical Appendix Table 2). Many PHLs might already have the computational resources needed to perform these bioinformatic analyses on a small number of samples in a reasonable amount of time. However, some basic command-line instructions would be needed to execute software. Numerous online resources, many of them free, will help novices learn the basics of the command-line interface. One such resource is the Biostar Handbook (https://www.biostarhandbook.com). This online document and e-book is an excellent resource that introduces bioinformatics and covers all of the major areas of focus in bioinformatics, including a crash course in the command-line interface.

## High-end Desktop Hardware

Computers with an increased number of processing cores, more RAM, and more storage space than the typical desktop or laptop computer will allow PHLs to perform all the analyses described here as well as more advanced and computationally intensive analyses (Figure). High-end desktops are relatively inexpensive to purchase, and it might be possible to upgrade desktops a PHL already has. All the analyses we describe here were performed at UPHL on an Apple iMac equipped with a single 3.2-GHz Intel Core i5 processor, 32 GB of RAM, and 2 TB of storage space (Technical Appendix Table 2). For 10 isolates, the analyses took ≈5 days to complete. Theoretically, the number of isolates that could be analyzed can be increased to up to hundreds of isolates on a similar high-end desktop computer; however, the amount of time to perform these analyses would also increase substantially.

## Beyond High-end Desktop Hardware

With a high-end Linux-based workstation (http://www.linux.org) and a network-attached storage array, several hundred genomes can be analyzed in a reasonable timeframe (Figure). At UPHL, we invested in a high-end Hewlett-Packard workstation (HP, Inc., Palo Alto, CA, USA) with four 3.0-GHz Intel Xeon processors (Intel Corp., Santa Clara, CA, USA), each 3.0 GHz with 12 processing cores; 256 GB of RAM; and a Synology network-attached storage array (Synology, Inc., Taipei, Taiwan) with 24 TB of storage (Technical Appendix Table 2). With such a system and bioinformatics personnel in place, hundreds of genomes can be generated and analyzed in 2–3 days, providing near real-time results for disease outbreak surveillance and monitoring. In addition to high-end computer hardware, experienced personnel are needed to deploy, maintain, curate, and automate bioinformatics pipelines (i.e., bioinformaticians). To take full advantage of computational resources, programs should be automated and linked together so that as data are generated by the sequencer, they are automatically added to the bioinformatics pipelines.

## Discussion

With NGS becoming more and more important for public health laboratories, the need for bioinformatic analyses in greatly increasing. Unfortunately, the pace of WGS implementation is far outpacing the number of bioinformaticians being hired to work in PHLs and, understandably, not all PHLs will have the need, desire, or financial capacity to hire a full-time bioinformatician. The objective of this perspective is to show that bioinformatic analyses can be performed on everything from a simple laptop to a high-end Linux workstation and the user can have little to no experience in bioinformatics or can be a full-fledged bioinformatician. As the volume of sequencing data increases, the ability to connect phenotype to genotype becomes a reality. Knowing a priori that a microorganism is likely to be resistant to antimicrobial drugs or could be a highly virulent strain would greatly improve patient outcomes, improve outbreak surveillance, and help prioritize resources to combat outbreaks. By using molecular evolutionary analyses, PHLs can investigate the evolution of antimicrobial-resistance genes to track in near real-time mutations that are linked to newly acquired resistance genes or novel mutations that result in resistance. 

NGS has the potential to revolutionize public health. NGS is not only replacing PFGE, but has the potential to replace traditional culture-based testing as well. Culture-independent diagnostic testing though metagenomic sequencing and analysis has the ability to quickly identify pathogens without applying any type of selection.

Technical Appendix Software and hardware used in the bioinformatic pipeline implemented at Utah Public Health Laboratory.
